# Parietal rTMS Induced Changes in Cortical Excitability in Patients With Minimally Conscious State Using TMS‐EEG

**DOI:** 10.1111/cns.70583

**Published:** 2025-09-04

**Authors:** Ye Zhang, Xiaoping Wan, Yong Wang, Xiaoli Li, Jubao Du, Xiao Yan, Zilong Zhu, Yanhua Li, Weiqun Song

**Affiliations:** ^1^ Department of Rehabilitation Medicine, Xuan Wu Hospital Capital Medical University Beijing China; ^2^ Department of Rehabilitation Medicine, Zhujiang Hospital, Southern Medical University Guangzhou China; ^3^ State Key Laboratory of Cognitive Neuroscience and Learning & IDG/McGovern Institute for Brain Research Beijing Normal University Beijing China

**Keywords:** coma recovery scale‐revised, minimally conscious states, repetitive transcranial magnetic stimulation, TMS‐EEG

## Abstract

**Objective:**

To verify the effectiveness of the parietal repetitive transcranial magnetic stimulation (rTMS) and take advantage of TMS‐EEG to assess cortical excitability in patients with minimally conscious states (MCS).

**Methods:**

We enrolled 10 MCS patients who received 10 sessions of 10 Hz rTMS on the parietal cortex for 10 consecutive days and then 10 days of sham stimulation after a 14‐day wash‐out period. The Coma Recovery Scale‐Revised (CRS‐R) and TMS‐EEG were used to assess the levels of consciousness and cortical excitability before and after active and sham stimulation, respectively.

**Results:**

Compared to sham stimulation, the CRS‐R revealed a significant improvement after receiving active rTMS. The amplitude of components of TMS‐evoked potential after active stimulation significantly increased at 30, 60, and 180 ms, respectively; global brain power increased significantly. The PCIst values had a significant difference after active rTMS stimulation, and the CRS‐R scores showed a significant correlation with PCIst values before and after active stimulation.

**Conclusion:**

High‐frequency rTMS on the parietal cortex has therapeutic efficacy and can improve cortical excitability in MCS patients.

**Significance:**

Our results provide preliminary evidence to support the use of the parietal area as a potential therapeutic target for MCS patients in clinical practice.

## Graphical abstract

CRS‐R assessments and TMS‐EEG measurements indicated that high‐frequency rTMS targeting the parietal cortex may have therapeutic potential by enhancing cortical excitability in MCS patients.

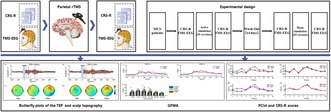

**Abbreviations**
GMFA: Global mean field amplitude
IIT: Integrated information theory
MCS: Minimally conscious state
PCI: Perturbation complex index
RMT: Resting motor threshold
UWS: Unresponsive wakefulness syndrome

## 1 Introduction

The term prolonged disorders of consciousness (pDoCs) refers to a pathological state of altered consciousness for at least 4 weeks caused by severe brain injury regions critical for initiation and maintenance of arousal. However, evidence‐based guidelines regarding the treatment of pDoCs are not available currently, while noninvasive brain stimulation techniques have been seen as potential experimental approaches to pDoCs treatment [1, 2, 3, 4].

A few clinical controlled trials using high‐frequency repetitive transcranial magnetic stimulation (rTMS) applied over the prefrontal [5, 6, 7, 8] and primary motor cortex (M1) [9, 10, 11] have shown promising results in minimally conscious state (MCS) patients. A prospective single‐blinded study showed that Coma Recovery Scale‐Revised (CRS‐R) scores were increased in all five MCS patients and four of 11 unresponsive wakefulness syndrome (UWS) patients after receiving 20 sessions of 10 Hz rTMS on the left dorsolateral prefrontal cortex (DLPFC) [6]. The electrophysiological data showed that rTMS decreased low‐frequency band power and increased high‐frequency band power, and induced significant changes in effective connectivity in MCS patients [7, 8]. The patients with more preserved alpha power and a significant reduction in the delta band induced by 20 Hz rTMS on the left DLPFC were likely to benefit in terms of therapeutic consciousness [12]. Furthermore, transcranial magnetic stimulation combined with electroencephalography (TMS‐EEG) might be an efficient assessment tool to evaluate the changes in cortical excitability by rTMS, and a significant increase in cortical excitability after 20 sessions of 10 Hz rTMS on the left DLPFC has been reported in an MCS patient [13]. High‐frequency rTMS over the left M1 has shown significant increases in peak systolic velocity and mean flow velocity of the left middle cerebral artery in MCS patients [9]. Although some studies could not provide sufficient evidence of the therapeutic effect of high‐frequency rTMS over the left M1 in MCS patients at the group level, results have indicated that MCS patients shortly after brain injury might possibly benefit from rTMS shortly after brain injury [10, 11].

Theories regarding the biological and physical basis of consciousness have recently been proposed. Integrated information theory (IIT) is one of the most important consciousness theories [14]. In contrast to global workspace theory (GWT), IIT primarily links consciousness with posterior cortical areas, partly because these areas exhibit neuroanatomical properties that are supposedly well suited to generate high levels of integrated information [14]. The posteromedial parietal areas, together with the medial frontal, anterior cingulate, and lateral parietal cortices, are more active when the brain is engaged in internal monitoring and in processing information related to one's self. The parietal lobe, as a key component of the posterior cortex, is implicated in various higher order cognitive functions [15] and has garnered growing interest as a potential target for noninvasive neuromodulation. Several studies have found that stimulating the posterior parietal cortex might influence cortico‐cortical and cortico‐thalamic connectivity, which are both damaged in patients with pDoCs [16]. Another study showed that 10 Hz rTMS over the left posterior cingulate cortex (PPC) significantly improved the functional recovery in UWS patients [17]. Our recent studies using event‐related potential (ERP) and EEG microstates demonstrated that parietal rTMS improves P300 amplitude and induces brain activity dynamics and may enhance frontal activity by improving frontal–parietal connectivity in pDoCs [18, 19]. However, the underlying electrophysiological evidence supporting the therapeutic efficacy of high‐frequency rTMS on parietal cortex for consciousness improvement in MCS patients has not been firmly established.

We, therefore, used TMS‐EEG to assess the cortical excitability of MCS patients treated with high‐frequency rTMS on the parietal cortex, comparing before and after both active and sham stimulation. We aimed to demonstrate the effectiveness of rTMS on the parietal cortex on pDoCs, which will support the use of posterior parietal as a potential therapeutic target for MCS patients in clinical practice.

## 2 Methods

### 2.1 Patients

We prospectively enrolled medically stable MCS patients hospitalized in the Department of Rehabilitation Medicine at Xuan Wu Hospital, Beijing, China, between February 2022 and March 2023. The inclusion criteria were as follows: (1) age18 to 80 years; (2) clinical diagnosis of MCS according to diagnostic criteria by CRS‐R; (3) time from onset of more than 4 weeks; (4) anoxic, traumatic, or vascular (i.e., hemorrhagic or ischemic) etiology; (5) MRI used to verify the integrity of the parietal cortex. The exclusion criteria were as follows: (1) previous history of neurological disease before coma; (2) patients with a metallic cerebral implant, pacemaker, or neurostimulator; (3) patients undergoing other treatments or drugs that modify cortical excitability; and (4) coexisting epilepsy or frequent spontaneous movements. During the experiment, all the patients underwent the basic rehabilitation program and medical treatment during the hospital stay.

### 2.2 Ethics Statement

This study was conducted in accordance with the ethical standards outlined in the Declaration of Helsinki (as revised in Brazil, 2013). Ethical approval was obtained from the Ethics Committee of Xuanwu Hospital, Capital Medical University (Approval No. 2022[022]). Written informed consent was acquired from the legally authorized representative of the patients prior to their inclusion in the study. The study was registered with ClinicalTrials.gov (NCT05682248). Patient anonymity and confidentiality were strictly maintained, and no identifiable information is disclosed in this manuscript.

### 2.3 Experimental Design and Stimulation Protocol

**FIGURE 1:**
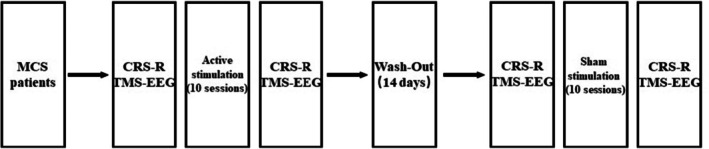
A flowchart of the experimental design.

The experimental design is shown in Figure 1. All the patients first received 10 sessions of 10 Hz rTMS on the parietal cortex for 10 consecutive days and then 10 days of sham stimulation after a wash‐out period of approximately 14 days (long enough for the effects to disappear) [17]. All the eligible MCS patients underwent clinical and TMS‐EEG evaluation by blinded assessors at four time points: (1) before the first session of active rTMS stimulation (A‐before); (2) immediately after the 10 active sessions (A‐after); (3) before sham sessions (S‐before); and (4) immediately after the 10 sham sessions (S‐after).

**FIGURE 2:**
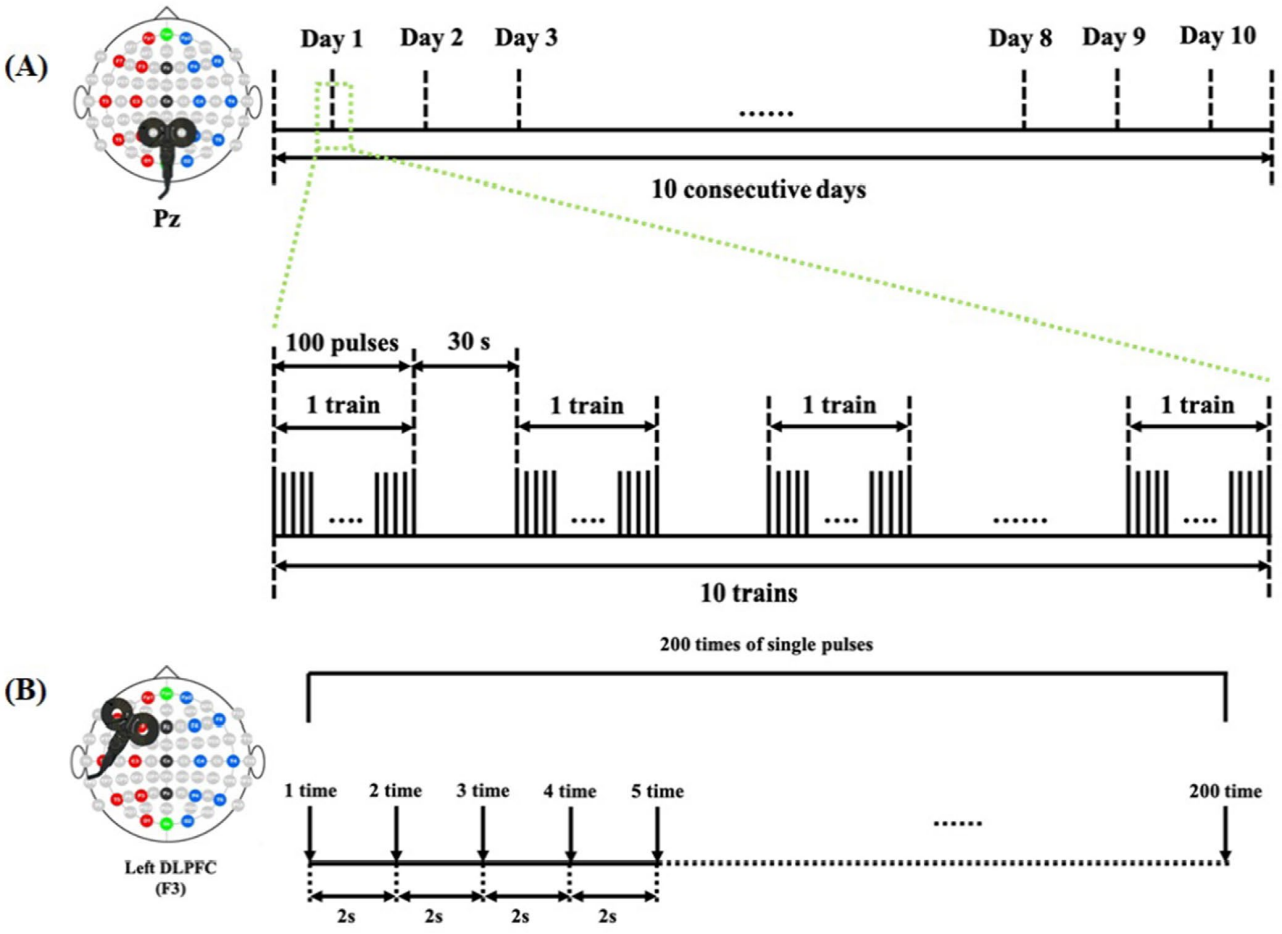
The active rTMS (A) and evaluation of TMS‐EEG (B) protocols.

During active rTMS or sham stimulation, patients received one session of stimulation each day for 10 consecutive days. Stimulation intensity varied across this experiment and was determined according to the resting motor threshold (RMT), identified as the minimum output intensity that induced a muscle contraction in which at least 5 out of 10 of the contractions had an amplitude > 50 μV peak‐to‐peak in the relaxed right abductor pollicis brevis [20]. Daily intervention sessions consisted of 1000 pulses (10 Hz) at an intensity of 90% RMT. The stimulation of one session included 10 trains, with each train lasting 10 and a 30 s inter‐train pause (Figure 2A) [18, 19]. The rise time of the magnetic monophasic stimulus was approximately 100 μs and time to zero was approximately 800 μs. The patients were seated in a semireclined position on the bed, and each session lasted approximately 10 min. TMS pulses were delivered via a Magstim R^2^ stimulator with a 70 mm figure‐of‐eight coil (Magstim Company Limited, Whitland, UK) placed tangentially onto the scalp over the parietal cortex. An EEG cap marked with the International 10–20 EEG System was used to identify the Pz (the parietal area) stimulation site. The rTMS treatment was administered according to the safety guidelines [21]. A specially designed figure‐of‐eight coil that does not produce a magnetic field but can mimic the acoustic artifact of rTMS stimulation was used for the sham treatment.

### 2.4 Clinical Evaluation

After being familiar with the ward environment for3 to 4 days, the patients underwent repeated CRS‐R behavioral assessments performed by the same two trained and experienced blinded assessors before the first and after the last session of active or sham rTMS. They performed separate assessments and then reached a consensus [22]. The CRS‐R assessment was conducted between 8 and 10 am Beijing time. This measure contained 23 hierarchically arranged items that comprise six subscales addressing arousal and auditory, visual, motor, oromotor/verbal, and communication functions. Responders were defined as a patient demonstrating at least one new sign of consciousness following stimulation. Conversely, nonresponders were defined by CRS‐R scores that were either stable or decreased after stimulation.

### 2.5 TMS‐EEG Evaluation

We used a Magstim‐Rapid^2^ stimulator (Magstim Company Ltd., London, UK), and a 70‐mm air‐cooled figure‐of‐eight focal coil connected to the stimulator to deliver TMS pulses. MR‐compatible BrainAmp amplifiers (Brain Products, Germany) were used to record EEG data from 64 channels in the international 10–20 system positions, with a special hardware design (bandwidth: DC, 1000 Hz; large recording range, 16 bits; and high sampling rate, 2500 Hz) to avoid the interference of the TMS pulse and collect instantaneous neural signals. During the experiment, the skin/electrode impedance was kept below 5 kΩ. As shown in Figure 2B, 200 single pulses were delivered to the patient's left dorsolateral prefrontal cortex [23, 24] and the EEG data were immediately recorded before the first and after the last session of active or sham rTMS. An EEG cap marked with the International 10–20 EEG System was used to identify the left dorsolateral prefrontal cortex (electrode cap F3 position) stimulation site. To activate the maximum cortical nerve activity, the TMS coil was held tangentially over the scalp, and the angle between the coil plane and the midline of the participant's brain was 45°. A layer of foam was placed between the coil and the stimulation target to reduce the volume of the click sound. To avoid contamination by auditory potentials evoked by the click associated with the TMS discharge, the patients wore earplugs continuously playing a masking noise. If the patient showed signs of sleepiness or was clenching their teeth or sweating, the CRS‐R arousal facilitation protocol was applied, or the experiment was suspended.

### 2.6 TMS‐EEG Pre‐Process

The pre‐process of the TMS‐EEG data was performed with scripts and functions from EEGLAB (v. 13.4.4b) and the TESA plugin for EEGLAB on the MATLAB platform v. 2020a (MathWorks, Natick, MA). Firstly, the original data were imported and the electrode position configured. Secondly, the TMS pulses were detected and marked. The EEG data were segmented into epochs (−1000 to 1000 ms around the TMS pulse) and baseline corrected (−500 to −110 ms). Thirdly, the TMS‐EEG trials containing noise, muscle activity, or eye movements were detected and rejected. Fourthly, the TMS‐EEG data were down‐sampled (1000 Hz) and the band pass filtered by a second‐order Butterworth filter (1–80 Hz). Lastly, ICA was performed to remove independent components containing large residual TMS‐evoked artifacts and artifacts related to eye movements, blinks, and muscular activity [24]. The TMS‐EEG data were re‐referenced to the average of all channels.

### 2.7 TMS‐EEG Analysis

We computed the average of all artifact‐free TMS‐EEG trials for further analysis. Globally, we drew butterfly plots of TEP and calculated the global mean field amplitude (GMFA) to capture dynamic variation of amplitudes across all electrodes. The GMFP is used to represent the standard deviation of TEP at a given time point and is calculated as follows:

$$\text{GMFA}\left(t\right)=\sqrt{\left[\frac{\sum_{i}^{k}{\left(V_{i}\left(t\right)-V_{\text{mean}}\left(t\right)\right)}^{2}}{k}\right]}$$

Where k is the number of electrodes, $V_{i}$ is the voltage of electrode i, and $V_{\text{mean}}$ is the average of the voltages of all electrodes.

For local TEP analysis, we drew three TMS‐evoked local potentials, including the frontal region (F7, F5, F3, F1, Fz, F2, F4, F6, and F8), the central region (T7, C5, C3, C1, Cz, C2, C4, C6, and T8), and the parietal region (P7, P5, P3, P1, Pz, P2, P4, P6, and P8). The mean and variance of TMS‐evoked local potentials were calculated for the statistical analysis.

In addition, we calculated the fast PCI (PCIst) used to characterize the time domain complexity of TEP [25]. As for TEP data A (c, t), c is the number of TEP channels, and t is the number of TEP time samples. First, we used singular value decomposition to obtain principal components of the signal as follows:

$$Ax_{i}=\lambda_{i}x_{i}$$

Second, we used recurrence quantification analysis to quantify the amplitude changes of single principal components before and after TMS, and obtain a distance matrix, $D_{j,k}\left(\text{before}\right)$ and $D_{j,k}\left(\text{after}\right)$, as follows:

$$D_{j,k}\left(\text{before}\right)=\left\|Ax_{i}\left(j\right)-Ax_{i}\left(k\right)\right\|$$

$$D_{j,k}\left(\text{after}\right)=\left\|Ax_{i}\left(j\right)-Ax_{i}\left(k\right)\right\|$$

Third, we binarized the matrix $D_{j,k}\left(\text{before}\right)$ and $D_{j,k}\left(\text{after}\right)$ to obtain a transition matrix, $T_{j,k}\left(\text{before}\right)$ and $T_{j,k}\left(\text{after}\right)$. The difference between the two transition matrixes was defined as time domain complexity of a principal component signal $P{\mathrm{TC}}_{i}$.

$${\mathrm{PTC}}_{i}=\frac{\mathrm{sum}\left(T_{j,k}\left(\text{after}\right)\right)-\mathrm{sum}\left(T_{j,k}\left(\text{before}\right)\right)}{\text{Tbefore}*\text{Tbefore}}$$

Last, PCIst was defined as the sum of $P{\mathrm{TC}}_{i}$ of all principal component signals.

### 2.8 Statistical Analysis

The data were analyzed using SPSS version 26.0 (SPSS, Chicago, IL). Paired t or Mann–Whitney U tests were used to compare the differences in CRS‐R scores, TEP, GMFA, and PCIst values between active rTMS and sham stimulation. The two variables are the difference in CRS‐R scores, TEP, GMFA, and PCIst values before and after active/sham stimulation as a percentage before active/sham stimulation, respectively. A Spearman correlation was performed to measure the correlation between PCIst and CRS‐R. P‐values < 0.05 were considered statistically significant.

## 3 Results

### 3.1 Enrollment and Characteristics of the Patients

A total of 12 MCS patients were screened, of whom 2 were excluded due to not meeting the age criteria. Ten MCS patients were enrolled. All patients underwent 10 days of active rTMS stimulation and then 10 days of sham stimulation after a wash‐out period of approximately 14 days. The demographic and clinical characteristics of the MCS patients are shown in Table 1. No significant side effects of rTMS were observed in this study.

**TABLE 1:** General conditions of MCS patients.

	Gender	Age (years)	Course (months)	Etiology	CRS‐R of baseline
MCS‐1	Female	53	6	Hemorrhagic	10 (2–3–2‐1‐0‐2)
MCS‐2	Female	65	5	Hemorrhagic	8 (2–2–2‐0‐0‐2)
MCS‐3	Male	32	4	Traumatic	8 (2–3–0‐0‐1‐2)
MCS‐4	Female	18	3	Traumatic	11 (2–3–3‐1‐0‐2)
MCS‐5	Female	18	7	Traumatic	17 (4–4–5‐2‐0‐2)
MCS‐6	Male	43	2	Traumatic	18 (3–4–5‐2‐1‐3)
MCS‐7	Female	70	10	Hemorrhagic	12 (3–3–2‐2‐0‐2)
MCS‐8	Male	55	4	Ischemic	12 (2–3–3‐2‐0‐2)
MCS‐9	Male	62	5	Ischemic	8 (2–2–2‐0‐0‐2)
MCS‐10	Female	35	3	Traumatic	9 (2–3–2‐0‐0‐2)

### 3.2 Clinical Measure Outcomes

The CRS‐R scores before and after active and sham rTMS stimulation are shown in Table 2. The total CRS‐R scores of all the MCS patients increased after 10 days of active rTMS stimulation, whereas most of the scores after sham stimulation remained the same (one increased; two decreased). The results revealed that, after active rTMS stimulation, a significant improvement was observed (*p* = 0.004), but no significant difference was observed after sham stimulation.

**TABLE 2:** CRS‐R scores of MCS patients before and after active rTMS and sham stimulation.

	A‐before	A‐after	responder/nonresponder	S‐before	S‐after	responder/nonresponder
MCS‐1	10 (2–3–2‐1‐0‐2)	12 (3–3–2‐1‐0‐3)	responder	12 (3–3–2‐2‐0‐2)	12 (3–3–2‐2‐0‐2)	nonresponder
MCS‐2	8 (2–2–2‐0‐0‐2)	14 (2–4–5‐1‐0‐2)	responder	15 (2–4–5‐2‐0‐2)	15 (2–4–5‐2‐0‐2)	nonresponder
MCS‐3	8 (2–3–0‐0‐1‐2)	14 (3–3–5‐1‐0‐2)	responder	12 (2–3–4‐1‐0‐2)	13 (2–3–5‐1‐0‐2)	nonresponder
MCS‐4	11 (2–3–3‐1‐0‐2)	12 (2–3–3‐2‐0‐2)	nonresponder	12 (2–3–3‐2‐0‐2)	12 (2–3–3‐2‐0‐2)	nonresponder
MCS‐5	17 (4–4–5‐2‐0‐2)	19 (4–4–6‐2‐0‐3)	responder	19 (4–4–6‐2‐0‐3)	19 (4–4–6‐2‐0‐3)	nonresponder
MCS‐6	18 (3–4–5‐2‐1‐3)	23 (4–5–6‐3‐2‐3)	responder	23 (4–5–6‐3‐2‐3)	22 (4–5–6‐3‐2‐2)	nonresponder
MCS‐7	12 (3–3–2‐2‐0‐2)	14 (3–3–4‐2‐0‐2)	responder	14 (3–3–4‐2‐0‐2)	14 (3–3–4‐2‐0‐2)	nonresponder
MCS‐8	12 (2–3–3‐2‐0‐2)	13 (3–3–3‐2‐0‐2)	responder	13 (3–3–3‐2‐0‐2)	12 (2–3–3‐2‐0‐2)	nonresponder
MCS‐9	8 (2–2–2‐0‐0‐2)	10 (3–2–3‐0‐0‐2)	responder	10 (3–2–3‐0‐0‐2)	10 (3–2–3‐0‐0‐2)	nonresponder
MCS‐10	9 (2–3–2‐0‐0‐2)	10 (2–3–2‐1‐0‐2)	nonresponder	10 (2–3–2‐1‐0‐2)	10 (2–3–2‐1‐0‐2)	nonresponder

*Note:* A‐before: before active rTMS stimulation, A‐after: after active rTMS stimulation, S‐before: before sham stimulation, S‐after: after sham stimulation.

Regarding the single subject, eight patients gained new signs of consciousness following active rTMS stimulation and were defined as rTMS responders, but no responders were identified after sham stimulation. Six patients (MCS2, MCS3, MCS5, MCS6, MCS7 and MCS9) improved in the motor subscore (two patients gained functional object use and emerged from the MCS), and four patients improved in the auditory subscore (MCS1, MCS3, MCS8 and MCS9). Furthermore, two patients (MCS4 and MCS10) showed improvement in oromotor/verbal function but did not gain any signs of consciousness. After sham stimulation, two patients' CRS‐R scores decreased (MCS6 and MCS8). One patient (MCS3) improved in the motor subscore, but did not show more conscious behaviors.

### 3.3 EEG Measure Outcomes

#### 3.3.1 TEP

**FIGURE 3:**
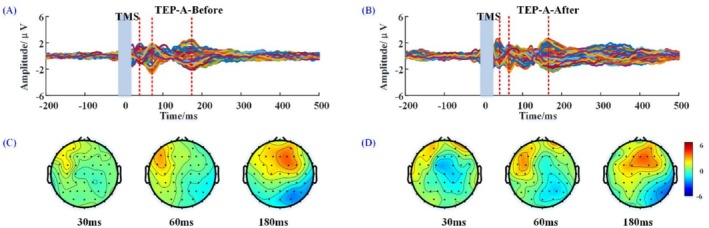
Butterfly plots of the TEP (A, B) and scalp topography (C, D) at 30, 60 and 180 ms before and after active rTMS. A‐Before: Before active rTMS stimulation; A‐after: After active rTMS stimulation.

The butterfly plots of the TEP before and after active rTMS are shown in Figure 3. The TEP after active stimulation showed more channel response activity and were more complex than before, with bigger amplitudes of fluctuation. Compared with sham rTMS stimulation, the amplitude of components of TEP after active stimulation at 30 ms (0.35 ± 0.17 μV), 60 ms (0.14 ± 0.28 μV), and 180 ms (1.06 ± 0.29 μV) were significantly increased to 1.31 ± 0.21 μV (*p* = 0.005), 0.88 ± 0.22 μV (*p* = 0.012), and 2.41 ± 0.27 μV (*p* = 0.001), respectively (Figure 3A,B). The scalp topography showed higher current intensity in the frontal cortex after receiving active stimulation at 30, 60, and 180 ms (Figure 3C,D).

**FIGURE 4:**
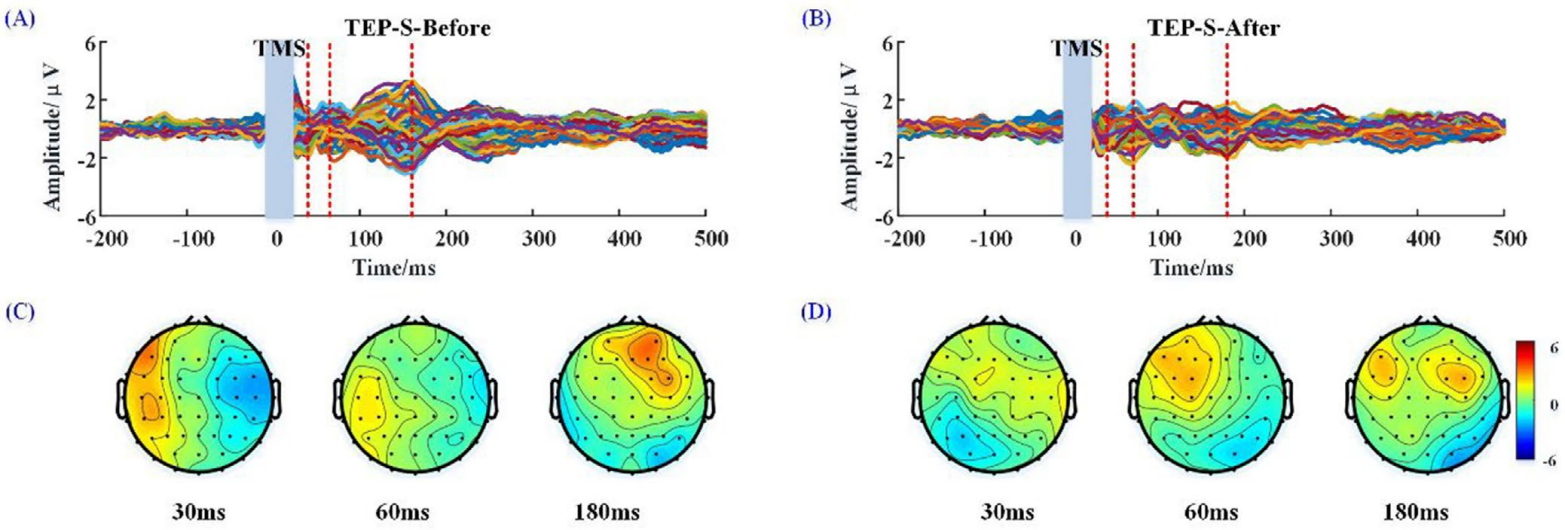
Butterfly plots of the TEP (A, B) and scalp topography (C, D) at 30, 60 and 180 ms before and after sham rTMS. S‐Before: Before sham rTMS stimulation; S‐after: After sham rTMS stimulation.

As for TEP before and after sham stimulation, the butterfly plots are shown in Figure 4. After sham stimulation, the amplitude of components of TEP at 30 ms (1.75 ± 0.22 μV) and 180 ms (2.80 ± 0.41 μV) decreased to 0.51 ± 0.22 μVand 0.92 ± 0.18 μV, respectively (Figure 4A,B). Correspondingly, the scalp topography showed lower current intensity in the frontal cortex after sham stimulation at 30 and 180 ms than before (Figure 4C,D).

#### 3.3.2 GMFA

**FIGURE 5:**
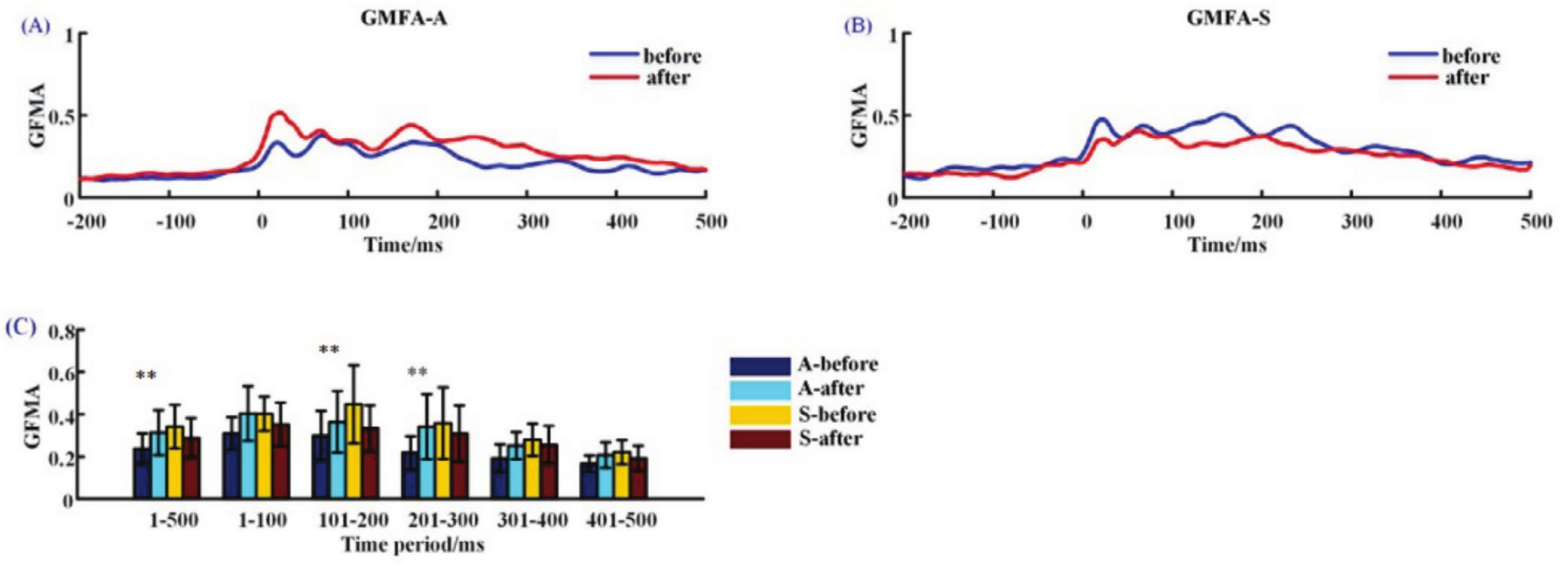
GMFA before (blue line) and after (red line) active rTMS (A) and sham stimulation (B). The mean GMFA in different time windows (C). A‐Before: Before active rTMS stimulation; A‐after: After active rTMS stimulation; S‐Before: Before sham rTMS stimulation; S‐after: After sham rTMS stimulation. **, *p* < 0.05.

The GMFA before and after active rTMS (Figure 5A) and sham stimulation (Figure 5B) are shown in Figure 5. After active rTMS stimulation, the global brain power increased significantly (*p* = 0.024), especially during the time window from 1 to 100 ms (*p* = 0.004) and from 201 to 300 ms (*p* = 0.030), while the GMFA increased, but not significantly, in other time periods. The global brain power after sham stimulation showed no significant changes (*p* > 0.05) (Figure 5C).

#### 3.3.3 PCIst

**FIGURE 6:**
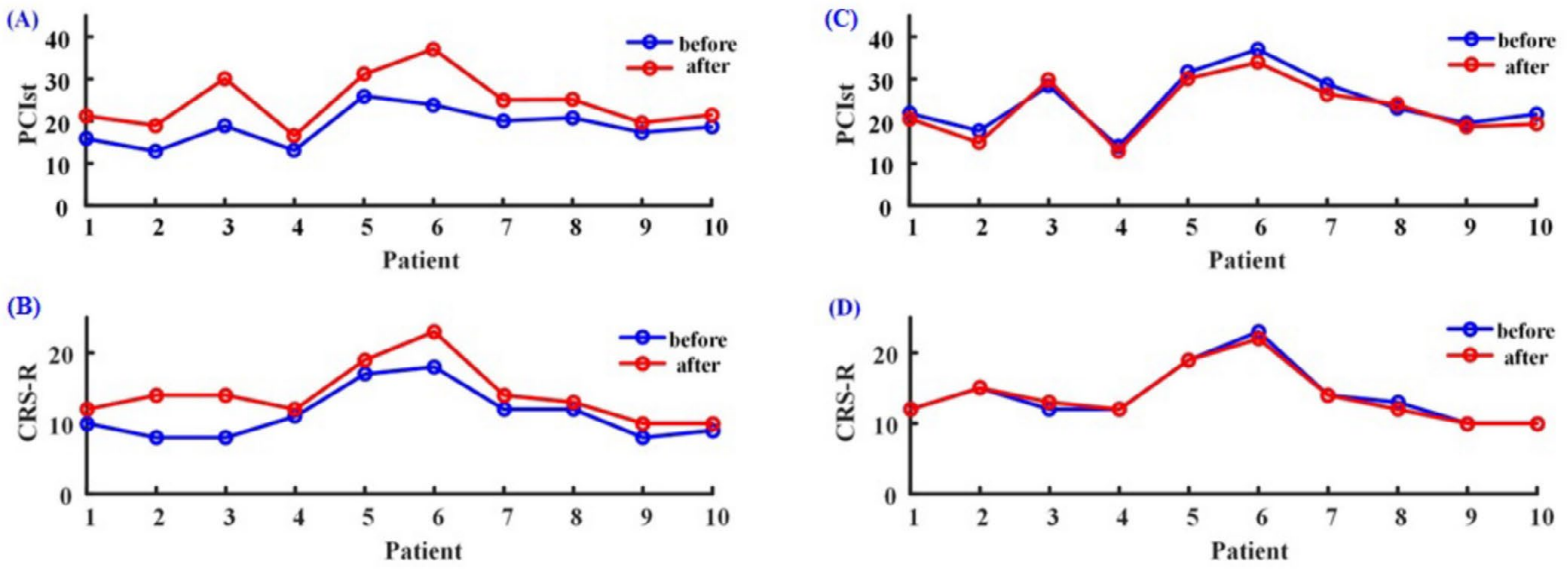
PCIst and CRS‐R scores before (blue line) and after (red line) active rTMS (A, C) and sham stimulation (B, D). PCIst, fast perturbation complex index; CRS‐R, Coma Recovery Scale‐Revised.

After active rTMS stimulation, the PCIst values and CRS‐R increased in all MCS patients and had a significant difference (*p* < 0.05), as shown in Figure 6A,B. After sham stimulation, the PCIst values and CRS‐R of patients showed no significant change (*p* > 0.05), as shown in Figure 6C,D.

### 3.4 Correlation Between PCIst and CRS‐R

**FIGURE 7:**
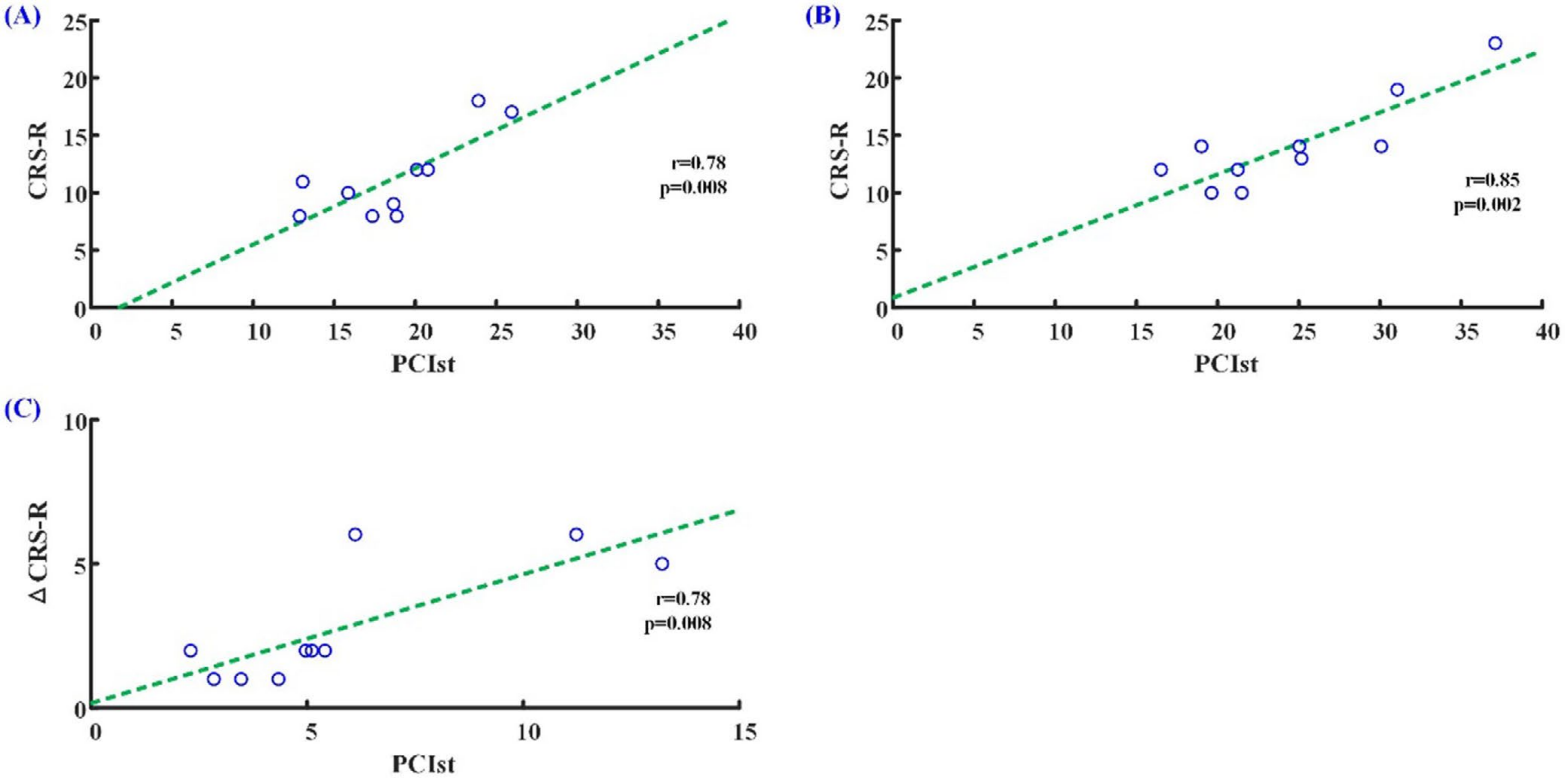
Correlation between CRS‐R scores and PCIst before (A) and after (B) active rTMS stimulation. The correlation between the differences of CRS‐R scores and the differences of PCIst values before and after active rTMS stimulation (C). PCIst, fast perturbation complex index; CRS‐R, Coma Recovery Scale‐Revised.

The CRS‐R scores showed significant correlation with PCIst values before (*r* = 0.82, *p* = 0.004) and after (*r* = 0.89, *p* < 0.001) active rTMS stimulation, respectively (Figure 7A,B). The differences in CRS‐R scores also showed significant correlation with PCIst values (*r* = 0.89, *p* < 0.001) before and after active rTMS stimulation (Figure 7C).

## 4 Discussion

Our data indicated that, compared with sham stimulation, high‐frequency rTMS on the parietal cortex can effectively modulate the amplitude of TEP, GFMA, and PCIst in MCS patients. More importantly, the changes in cortical excitability presented by the MCS patients showing clinical improvements in consciousness were consistent with the findings from the overall study. To the best of our knowledge, this is the first prospective investigation of whether high‐frequency rTMS on the parietal cortex affects cortical excitability in MCS patients by using TMS‐EEG.

The prefrontal cortex has extensive reciprocal connections to wake‐promoting centers in the brainstem and diencephalon and is, therefore, the most chosen stimulation site. However, this does not exclude the possibility that the recruitment of additional brain areas, such as the posterior cortical area proposed to be important for conscious thought, could be required to restore the complete spectrum of consciousness. The posterior cortical areas primarily represent sensory modalities and are most likely candidates for the phenomenological component of conscious experience [26]. The posterior cortex encompasses a “posterior hot zone” implicated in generating various conscious experiences, including vision, hearing, and touch [27, 28], providing direct evidence that posterior brain regions are critically involved in human consciousness. In particular, the parietal cortex is a main player in guiding the expansion of neural activity across brain areas [29]. Our results are uniquely valuable in identifying the posterior parietal area as a potential target for clinical intervention through causal research. Clinical and correlative data have been used to support the unique position for prefrontal and posterior cortices to modulate the level of consciousness [30]. Multifocal stimulation protocols including both the prefrontal and posterior parietal cortexes may be designed to target different parts of the consciousness network considering the diversity of patient brain injuries. A study showed that a single session of multifocal frontoparietal tDCS did not have behavioral effects in pDoCs at the group level [31]. Compared to tDCS, rTMS delivers more intense and focal stimulation, with its therapeutic effects further enhanced through repeated sessions. Our preliminary findings suggest that 10 Hz dual‐target rTMS, applied to both the prefrontal and parietal cortices, has therapeutic potential for improving behavioral outcomes and enhancing brain oscillatory activity in the frontal, central, and parietal regions in patients with MCS [32]. However, further studies are warranted to determine whether multifocal rTMS yields superior efficacy compared to single‐site stimulation and to identify optimal individualized stimulation parameters.

The role of the main player could be reflected in the changes of CRS‐R scores. Most patients experienced an increase in auditory, motor, and oromotor/verbal function after the active rTMS stimulation. This may be explained by the anatomy and the connections among cortexes [33]. The fibers originating from the precuneus (the corresponding site to Pz of the stimulation site in the parietal cortex) could extend to the temporal cortex where the auditory cortex is located, and extend to the premotor and supplementary areas which involve motor control and coordination. During prospective excitability, the amplitude of the TEP component increased and the GMFP underwent a significant increase after receiving active rTMS stimulation. Furthermore, tracing the current distribution of the scalp, the intensity was enhanced in the frontal cortex after active rTMS stimulation. As for connectivity, the PCIst also showed a significant increase. However, this phenomenon was not seen after receiving sham stimulation.

EEG studies have important implications for medicolegal decision‐making in pDoCs. The resting‐state EEG captures spontaneous activities of neuronal assemblies, while EEG responses combined with different external stimulations represent different neural pathways and circuits, which can reflect the ability of neural networks to integrate information [34]. TMS‐EEG has emerged as a powerful tool to noninvasively probe brain circuits in humans, allowing for the assessment of several cortical properties such as excitability and connectivity [35, 36]. In the DoC field, TMS‐EEG can also measure the effect of invasive and noninvasive brain stimulation [13, 24, 37, 38]. The perturbation complex index (PCI) has been proposed to quantify the temporal–spatial complexity of TEPs and reflect the ability to integrate information of the cortex, which is considered one of the key neurophysiological bases of human consciousness. However, the variety and complexity of EEG measures and analyses of PCI create obstacles to being a practical and widely applicable tool. In view of this, Comolatti et al. proposed a PCIst algorithm [25] which was reported as a fast and stable method to empirically estimate the complexity of brain responses to external stimulation [39]. Our results showed that the change trend of PCIst values was consistent with the CRS‐R score, which will further support the use of PCIst as a potential efficacy evaluation tool for pDoCs in clinical practice.

Brain connections are more than the mere transfer of signals between brain regions. Increasing evidence has revealed that brain connections also support the underlying mechanisms which determine the function of the brain and cognition [40]. Understanding how cortical connectivity recovers in pDoCs can inform neurobiological theories of consciousness and guide clinical investigations. Several studies have shown that the cortical reactivity and connectivity are preserved in most MCS patients, while they are also suppressed in UWS patients. TMS‐EEG plays an important role in mapping causal interactions between brain regions. Causality can be inferred by combining focal TMS stimulation with distant real‐time physiological recordings. These combinations are high on the causality continuum, as they employ targeted stimulation [41]. A TMS‐EEG study has demonstrated that the occurrence of OFF periods in cortical circuits impairs local causal interactions and further prevents the build‐up of global complexity in UWS patients [42]. Our results showed that some cerebral regions could be re‐excited by rTMS in MCS patients. The observed rTMS‐related behavioral improvements might be related to the more residual global complexity and connectivity in MCS compared to UWS.

There are several limitations in our study. Major drawbacks include the relatively small and heterogeneous (different etiologies and times post‐injury) sample of MCS patients. We only focus on MCS patients because they have more preserved information processing abilities than UWS patients, and a priority to recover [43]. We did not randomize the order of treatment between the patients due to limitations in clinical work. Thus, further sham‐controlled, randomized, crossover studies with larger sample sizes are needed to support our conclusions. Second, we did not use rTMS combined with the MRI neuronavigation system but instead used the 10–20 EEG System to identify the parietal area and left DLPFC in rTMS and TMS‐EEG procedures, respectively, which cannot ensure precise targeting. This method is clinical as it is less expensive and less complicated, and there are fewer hospitals equipped with a navigation system actually. Therefore, our results can provide direct guidance for rTMS treatment for MCS patients. Additionally, functional neuroimaging techniques were not used. Since an understanding of the neural correlates is crucial for effective therapeutic interventions, combining EEG with other neuroimaging methods, such as ^18^F‐fluorodeoxyglucose‐positron emission tomography (FDG‐PET) or functional magnetic resonance imaging (fMRI) should be applied to obtain a broader and more holistic evaluation of therapeutic efficacy in pDoCs in future studies. Several previous studies have also shown that not all pDoCs benefit from the rTMS procedure [17, 18]. Therefore, a prediction model should be developed to explore a reliable biomarker of responsiveness prior to the clinical application of rTMS. In addition, an optimal rTMS protocol is essential to achieve the targeted effects. Considering the unique positions of different brain regions to modulate levels of consciousness, further studies should employ a multifocal rTMS protocol based on patient‐tailored brain structures.

## 5 Conclusion

Our study indicated that high‐frequency rTMS on the parietal cortex can improve cortical excitability and show behavioral improvement in patients with MCS. The current study provides preliminary evidence to support the use of posterior parietal areas as a potential therapeutic target for MCS patients in clinical practice.

## Author Contributions

**Ye Zhang:** conceptualization, investigation, resources, methodology, writing original draft, writing review and editing, supervision. **Xiaoping Wan:** methodology, formal analysis, visualization. **Yong Wang:** methodology, formal analysis, visualization. **Xiaoli Li:** writing review and editing. **Jubao Du:** investigation, resources, writing review and editing. **Xiao Yan:** writingreview and editing. **Zilong Zhu:** writing review and editing. **Yanhua Li:** investigation, resources. **Weiqun Song:** conceptualization, writing original draft, writing review and editing, supervision.

## Disclosure

The authors have nothing to report.

## Conflicts of Interest

The authors declare no conflicts of interest.

## Data Availability

The data that support the findings of this study are available on request from the corresponding author. The data are not publicly available due to privacy or ethical restrictions.
